# Essential and Expendable: Migrant Domestic Workers and the COVID-19 Pandemic

**DOI:** 10.1177/00027642211000396

**Published:** 2021-09

**Authors:** Kritika Pandey, Rhacel Salazar Parreñas, Gianne Sheena Sabio

**Affiliations:** 1 University of Southern California, Los Angeles, CA, USA

**Keywords:** essential workers, elderly care, domestic work, migrant labor

## Abstract

In this article, we examine the effects of the COVID-19 pandemic on the labor conditions of domestic workers in the epicenter of the United States. We focus our analysis on the symbolic categorization of domestic work as “essential labor.” While domestic workers are lauded as heroes in public discourse, we argue that this symbolic recognition does not extend to material remuneration. Instead, we find that labor conditions better fit their categorization as *expendable essential workers*, meaning those whose essential labor is magnified during the pandemic but whose work remains materially undervalued. Data used in this article draw from observations of more than 30 hours of virtual town hall meetings on the pandemic hosted by migrant domestic worker advocacy groups in Los Angeles and New York.

Essential work^1^ refers to the labor of meeting basic needs for human survival and well-being. It includes a vast array of jobs entailing the provision of transportation, health care, food and agriculture, security, janitorial service, and domestic work (Lakoff, 2020). This type of work has become particularly magnified during the COVID-19 pandemic including in the epicenter of the United States, where the vast majority of essential workers are immigrants and people of color (Howes et al., 2012; Nunn et al., 2020). They include Southeast Asian refugees in meat processing plants (Mize & Swords, 2010), Latinx and Filipinx migrant domestic workers (Hondagneu-Sotelo, 2001; Parreñas, 2015), Mexican migrant farmworkers (Mize & Swords, 2010), and Filipinx migrants in the health industry (Nazareno, 2018; Ortiga, 2018).

According to anthropologist Andrew Lakoff (2020), the construction of an “essential workforce” is a key state strategy for managing national crises with particular individuals put at greater risk for the sake of the overall well-being of the population. Those put “at risk” tend to be undervalued workers. At a count of 48 million, essential workers comprise approximately 42% of the U.S. working population (Nunn et al., 2020). Indicating that occupations labeled as “essential work” are likely to be those considered low wage and low status, a report drawing from U.S. Department of Labor Statistics indicates essential workers to earn less than employees in other industries with those in California earning 13.9% less and in New York 24.8% less (McQuarrie, 2020). This is likewise the case for the estimated 2.2 million domestic workers in the United States, as they earn a median hourly wage of $12.01 as compared with $19.97 for other hourly wage workers (Wolfe et al., 2020).

This article addresses the valuation of essential work during the COVID-19 pandemic. It underscores the contradictory position of being symbolically categorized as essential but made to be treated as indispensable and undervalued workers. It examines this contradiction by looking at the case of domestic work. We focus on domestic workers because they are arguably at the forefront of the COVID-19 crisis due to their care for the vulnerable population of the elderly. In numerous discussions establishing the elderly as a high-risk group for COVID-19,^2^ little attention has been given to those who are paid to care for them, specifically the caregivers who tend to their needs in private homes, long-term care facilities, and nursing homes. The contradiction of being simultaneously essential and expendable is notably one not lost on domestic workers and their advocates, including Linda Oalican, the executive director of Damayan, a migrant work advocacy group serving the Filpinx community in New York City, who shares,

The rich people in the state of New York and over all the country have migrant workers working as their nannies, their babysitters, their housekeepers and as caregivers taking care of their parents . . . But all along we know that they see us as disposable labor, workers without rights. They can choose to get rid of us any time they want with little accountability.

Elaborating on this contradiction, this article documents as it examines the experience of being essential and yet expendable. It asks, how have working conditions for essential workers changed during the pandemic and how can we characterize such changes?

This article begins with a literature review on domestic work, establishing its historic devaluation as low-paid women’s work. It then proceeds with a description of our methods and data. Proceeding with our analysis, we then describe the labor conditions elicited by the pandemic for domestic workers: high risk of unemployment, demand for overtime work, unremunerated labor, unsafe workplaces, and minimal access to state benefits extended to other essential workers. Our findings do not only indicate the continued devaluation of domestic work but also establish the emergence of domestic workers as those who we can label as *expendable essential workers*, meaning those performing essential work that is magnified symbolically but devalued materially. Examining the significance of our findings, we end with a discussion of how the label of “essential work” can become a controlling mechanism used by employers to maximize labor.

## Literature Review

There is a general consensus among studies on domestic work of the devaluation of this labor due to first the historic and feudal roots of the occupation (Boris & Nadasen, 2008), second the culture of the workplace and the social construction of employers as “superior” (Rollins, 1985), and third the informal structure of the occupation (Glenn, 1986; Hondagneu-Sotelo, 2001; Romero, 2002). For these reasons, domestic work has historically been perceived as an unskilled occupation. In this article, we add to this discussion by illustrating how cultural constructions of domestic work, specifically labelling it as essential, form a fourth way of devaluing paid domestic work and can be utilized as a controlling mechanism for extracting uncompensated labor.

In the United States, the historic roots of domestic work can be traced to the concentration of African Americans in this occupation after their emancipation from slavery (Hunter, 1998). It is argued that the ghettoization of not only African Americans but also other women of color in the occupation led to the racialized exclusion of domestic work from labor protection (Boris & Nadasen, 2008; Glenn, 1986). In the United States, the devaluation of domestic work is reflected in their historic exclusion from labor protection. For example, domestic work had been excluded from the Fair Labor Standards Act (FLSA) of 1938, which extended the right to minimum wage and overtime protection. In 1974, Congress amended FLSA to extend to domestic workers but excluded those who provide “companionship services,” specifically referring to companionship extended to elderly persons or persons with illnesses, injuries, or disabilities. Yet, in 2015, the Department of Labor revisited its regulations and finally extended FLSA protection to those providing “companionship services” including live-in domestic workers. However, the legacy of devaluation endures as domestic work including eldercare remains among the lowest paid jobs in the United States (Shierholz, 2013).

The devaluation of domestic work can also be attributed to the culture of the workplace, which takes place not in a vacuum but in a larger societal context shaped by race, class, and gender hierarchies (Glenn, 1992). In the early 20th century, distance defined employer–employee relations as a stark division of labor marked the relationship between domestic workers and employers; the relegation of physically onerous and demeaning household labor, that is, the dirty work, to the former allowed the latter to embrace their domesticity (Palmer, 1991). According to Phyllis Palmer (1991), the division of labor in households distinguished “clean mistresses” and “dirty servants” as the more physically strenuous labor of the servant enabled the mistress to attain the markers of ideal femininity—fragility and cleanliness. By the late 20th century, distance eventually gave way to the cultivation of personal relationships in the household (Hondagneu-Sotelo, 2001; Rollins, 1985). This cultural shift blurred the status of the domestic worker as an employee and paved the way for employers to make demands that exceed the contractual obligation of the worker (Romero, 2002).

Last, scholars attribute the devaluation of domestic work to the structure of the occupation (Hondagneu-Sotelo, 2001). The informal structure as well as the isolation of the job pose a challenge to enforcing employment standards (Romero, 2002). Domestic workers have attempted to gain greater control of their job by countering the personalism expected by employers through the enforcement of distance, which they do by avoiding-live in work and becoming their own employer who charge households that hire them not an hourly wage but a rate per job (Romero, 2002). To counter the vulnerabilities engendered by the informal structure of the occupation, domestic workers and their advocates also turn to the law and the standardizing of their employment (Blackett, 2019). This is illustrated by their lobbying of the International Labour Organization for the successful passage of Convention 189, otherwise known as the Decent Work for Domestic Workers Convention (Fish, 2017). However, the difficulty of enforcing the law due to the private setting and informal nature of the occupation continues to haunt domestic workers (Bakan & Stasiulis, 1997; Hondagneu-Sotelo, 2001).

In this article, we build on previous research that establishes the devaluation of domestic work including elderly caregiving and examine how their symbolic recognition as “essential” shapes their labor. In doing so, we add a fourth way that domestic work gets devalued as we establish that the cultural construction of a job can be a controlling mechanism utilized by employers to maximize the labor of domestic workers. Indeed, other scholars have established this to be the case with the use of the discourse of “one of the family” as a tool for blurring the status of domestic workers as paid laborers, conflating their work with family obligation, and constructing domestic work to be a “labor of love” (Romero, 2002). In this article, we show that the labeling of domestic workers as “essential” likewise results in the magnified demand for their labor but not necessarily the betterment of their labor conditions.

## Data and Methodology

This article draws data from observations of virtual town hall meetings organized by two advocacy groups for Filipinx migrant workers based in the two cities worst-hit by the pandemic, Los Angeles and New York (Centers for Disease Control and Prevention, 2020). The two organizations we observed are Pilipino Workers Center (PWC) in Los Angeles and Damayan Migrant Workers Organization in New York. PWC and Damayan are nonprofit organizations that cater to low-wage Filipinx workers by providing labor rights education, workforce trainings, legal and immigration assistance, support for human trafficking survivors and victims of wage theft, and other related services. PWC and Damayan were established in 1997 and 2002, respectively, and maintain a membership base that is mostly composed of domestic workers.

In total, we observed more than 30 hours of virtual town halls for migrant caregivers hosted by the two advocacy organizations since March 2020. We analyzed 16 meetings that typically lasted two hours. In these town hall meetings, discussions specifically revolved around the challenges confronted by migrant domestic workers during the pandemic and what types of strategies they should develop to address them. The topics addressed include the general situation of domestic workers and how to avail of services and assistance including for instance what to do if terminated by employers (seven meetings); occupational safety and health concerns such as reducing the risk of infection (five meetings); housing, labor, and immigrant rights such as eligibility for state benefits of undocumented workers (three meetings); and last systemic racism in light of the Black Lives Matter movement (one meeting).

Meetings were typically facilitated by community organizers and featured a broad range of speakers that included staff of the organizations, workers providing testimonials, and a number of experts including medical professionals, public health specialists, psychologists, lawyers, and community advocates. Both advocacy groups hosted the meetings through a videoconferencing platform, while simultaneously broadcasting them via social media including Facebook and YouTube. In both channels, members and viewers shared their concerns, questions, and other experiences either verbally or by posting them as “comments” that were then read out by the meeting moderators. While a quantified profile of the participants cannot be ascertained given the cross-platform online format of the meetings, it can be gleaned from the verbal discussions and written interactions that many of them are female Filipinx who are working as domestic workers. Videos were publicly archived in the groups’ social media pages, allowing us to systematically review the conversations and identify patterns of risks, precarities, and dilemmas confronted by Filipinx migrant domestic workers. Our data specifically draw from testimonies and questions posed by domestic workers who participated in the town hall meetings. While these meetings were accessible to the public, we formally informed PWC and Damayan that we were observing their town halls for research purposes.^3^

The COVID-19 pandemic constrains the means by which we could collect data as it prevents us from interacting face-to-face with migrant domestic workers. It also limits the representation of our sample as we are unable to identify workers not tied to a migrant advocacy group. Traditionally, we would identify domestic workers by patronizing ethnic businesses, attending religious services, and participating in gatherings of migrant community groups. This limitation means that our data prevent us from making broader claims about the conditions of domestic work during the pandemic. For instance, we recognize that our research subjects are likely those facing workplace issues because they are those most likely to participate in a meeting of a community advocacy group. Another limitation, in contrast to other research methods such as interviews and focus group discussions, is that virtual participant observation does not allow us to establish the frequency or prevalence of the problems reported during the town hall meetings. We addressed this concern by triangulating our observation data with the organizations’ reported data about their members; in particular, we used findings from their informal surveys to validate and contextualize our observations. In light of these methodological constraints, this article is not making any claims about the universality of the problems facing domestic workers but is merely identifying various issues that have arisen during the COVID-19 pandemic.

## Essential and Expendable: Labor Conditions of Domestic Workers in the COVID-19 Pandemic

The situation of migrant domestic workers in the epicenters of Los Angeles and New York indicates that their categorization as essential workers does not reduce their workplace vulnerabilities. Instead, we find the COVID-19 pandemic aggravates the insecurities that other scholars have identified for domestic workers including inadequate compensation (Romero, 2002), forced labor (Bales & Soodalter, 2009), and lack of workplace safety (Nazareno et al., 2014). Yet, the pandemic does not only aggravate preexisting vulnerabilities, meaning those that have been identified in the literature, but also creates new workplace issues, including the precarity of unemployment and housing, both of which are risks that have yet to be associated with domestic work in previous studies.

### Preexisting Problems

Arlene Janer, a middle-aged Filipinx domestic worker, sits on the edge of her bed as she prepares to share her “Worker Testimony” in a virtual town hall meeting on the impacts of COVID-19. Embodying her experience is the aggravation of the various preexisting problems long-established in the literature in domestic work—unfair pay, coerced labor, and unsafe workplaces. With the exception of a handful of outsiders including researchers such as ourselves, Ms. Arlene^4^ speaks primarily to domestic workers in what is known as the Tri-State Region of the greater New York City area, which covers New York, New Jersey, and Connecticut. Ms. Arlene had been a live-out domestic worker for a wealthy family in New York City prior to the pandemic. She worked from 8 a.m. to 6 p.m. on Mondays to Fridays and returned home every night to her shared apartment in the neighborhood of Elmhurst, Queens, which is home to many Filipinx domestic workers.

Ms. Arlene shares that her life has been upended since the pandemic. It is no exaggeration to say that she has found herself burdened with greater work responsibilities, coerced into live-in work, denied a day off, inadequately compensated for her extra hours of work, and confronted with mental health issues from her isolation in the Hamptons, seaside communities in Long Island for affluent New York City residents. Since relocating, Ms. Arlene claims that her work hours have increased to 15 hours per day for seven days a week as her responsibilities have expanded to entail housecleaning and not only childcare. She feels that the pandemic gave her two choices—relocate and be overworked in the Hamptons or stay and become unemployed in New York City. The dilemmas of Ms. Arlene are not exclusive to her, but it is one that continuously resonated in town hall meetings.

#### Inadequate Compensation

During the pandemic, domestic workers confront inadequate compensation in two ways: the lack of sufficient severance on their termination or the lack of adequate material compensation for the added labor of those who continue to work. While Ms. Arlene has managed to keep her job during the pandemic, many of her peers have not as they have faced sudden termination without adequate compensation. The sudden termination of domestic workers is a long-standing problem that reflects the absence of job security in the domestic work industry (Hondagneu-Sotelo, 2001). It is one aggravated during the pandemic. A recent survey administered by the National Domestic Workers Alliance (NDWA) found that 72% of household workers lost their jobs since the pandemic (Tarrant, 2020). Those who did, found they had not been entitled to severance pay. It is for this reason that many have turned to the assistance of community advocacy groups for survival. Since the pandemic, organizations such as PWC and Damayan have distributed more than 400 boxes of groceries to domestic workers.

Substandard compensation is another problem confronted by domestic workers that has been magnified during the COVID-19 pandemic. Due to the informal arrangement of domestic work (Hondagneu-Sotelo, 2001; Romero, 2002), employers can easily turn their back on extending benefits to domestic workers including health insurance, overtime pay, and as mentioned earlier severance pay. Not surprisingly, domestic workers complained that employers are refusing to compensate them for the additional hours of service they are demanding during the pandemic, which now averages 70 hours as opposed to 50 hours per week prior to the pandemic. This includes Ms. Arlene, who shares, “What is sad is that they are not compensated fairly for long hours of their service just like me. Others are not paid at all for those extra hours of service.”

#### Coerced Labor

The public meetings and townhall discussions reiterate the indispensability of domestic workers as they simultaneously identify the aggravation of the preexisting vulnerability of coerced labor. First, domestic workers find themselves with the compulsion to work in order to survive regardless of work conditions or lack of support such as health insurance. In multiple public meetings, organizers asserted that workers feel compelled to work due to the risk of losing their income. Second, employers are increasingly demanding that workers switch to live-in employment. The pandemic puts workers under duress with this added pressure to acquiesce to the demands of the employers for the sake of protecting their livelihood.

According to testimonies and comments in the town hall meetings, the increased reliance of employers on domestic workers has not reduced power inequalities in the workplace. The heightened dependence of employers is illustrated for instance by the extension of a one-time US$4,000 bonus to Ms. Arlene. For her, this recognition is, however, merely symbolic because it fails to address the persistent structural inequalities in their relationship. Ms. Arlene would rather have her employers recognize her sacrifice of relocating to the Hamptons by revisiting their “no work, no pay” arrangement, which is a condition that they know has long been an issue for her. At the end, it is still employers determining the labor conditions and terms of compensation with little input from domestic workers during the pandemic. For this reason, the bonus extended to Ms. Arlene is rather exceptional and not an experience shared by most of her peers. Other issues they have long faced likewise remain unaddressed. This includes the inadequacy of occupational health and safety (Nazareno et al., 2014).

#### Unsafe Workplace

As we all know COVID-19 makes our lives uncertain, not just caregivers but everybody. Where I work, it is a skilled nursing facility. It is supposed to be a five-star facility but what happens is we were not given proper protective gear . . . as soon as it was acknowledged that there is such a virus which is very dangerous. It was only two weeks ago, when I was still working that we learned that there were already residents in our facilities that had tested positive after testing, and I began fearing for my safety—especially as I am at the age where I am vulnerable and also I have preexisting conditions. I informed my agency that I need to take off work voluntarily and take care of myself because how can I take care of my patients and residents if I cannot take care of myself. (Lee Plaza, a private caregiver, in a nursing facility)

Lee’s experience at her workplace, not unlike many of her peers, lays bare the fact that domestic workers are disposable. During the pandemic, their physical and mental health are at risk. This is due in large part to their unsafe work environments whether in a nursing facility or a private home. The lack of safety in their workplace is the ultimate expression of their daily struggles with exploitation and persistent marginalization. This observation is not lost among the workers themselves. As Linda comments, “This war against this invisible enemy, this pandemic has exposed how we are treated, as disposable lives in America.”

Aggravating the risks emerging from COVID-19 is their exclusion from various legal provisions. Occupational Safety and Health Administration (OSHA) laws, which cover most private sector employers and workers across the United States are not necessarily inclusive of domestic workers.^5^ While some relief is provided to workers in private nursing homes or assisted living facilities, this is not the case for those in private households. Even in cases where OSHA laws can support worker rights, getting these enforced in the workplace is still a struggle. As an organizer explained, “Unfortunately, OSHA is not an agency that has a very strong enforcement arm or history. It has been underresourced for years.” For domestic workers, the lack of workplace safety is one that has been aggravated during the pandemic due to the risk of infection among health care workers. It is for this reason that domestic workers in town hall meetings shared how their mental health and well-being is compromised as the physical risk of exposure puts a strain on their psychological well-being.

From janitors to bus drivers, essential workers are those most vulnerable to the physical risk of exposure. One could argue that essential workers in the informal labor market are made more vulnerable by their minimal access to public support. This is the case for domestic workers, especially those with undocumented immigration status. As Linda reflected, “Even the government, the American society is struggling to get the PPEs to protect their health workers, the vulnerable section of their society. Who is thinking about us, right?” It is questions like these that explain why migrant advocacy groups find themselves with a greater burden to provide services to domestic workers during this pandemic, hosting online meetings with medical doctors, psychologists, lawyers, and meditation specialists to address the different problems that have arisen for domestic workers. As we illustrated in this section, many of these problems are preexisting yet heightened by the pandemic. In the next section, we identify the new problems that have arisen from COVID-19.

### New Problems

An outspoken advocate in Southern California is Lolita Lledo, the associate director of PWC. From job opportunities to workplace concerns, Lolita is a one-stop-shop for everything that concerns domestic workers in the region. For this reason, many in the community go to her if facing a problem at work. This includes the caregiver “Tony,” who found himself homeless during the COVID-19 pandemic. Lolita was shocked to learn of this new problem, as homelessness is an issue she has never associated with domestic work. Sharing her outrage, she took to social media to express her consternation and plea for assistance,

Tony is a caregiver in Los Angeles and was told that his consumer/patient^6^ was positive with coronavirus. He was sent home and ordered to self-isolate himself. His roommates begged him not to enter their shared room. So out of desperation, he decided to pitch a tent in the backyard of their apartment. He became a homeless caregiver and PWC is doing a fund drive to give Tony and other caregivers who are and will be caught in this situation a decent room that they can stay while waiting for the result of the corona test. Any help will be greatly appreciated.

Tony, a fairly young caregiver, had a patient test positive for COVID-19. Soon after, his patient’s family told him to leave his job, go home, and quarantine himself. Like many other migrant Filipinx domestic workers, Tony lives in Historic Filipino Town, an area immediately west of Downtown Los Angeles, and shares a room in an apartment with several people. As a room in a shared apartment typically costs $1000 in this neighborhood, it is not uncommon for two to four migrants to share one room in order to offset the prohibitive cost of housing in the area. His roommates heard the news and before he could set foot in their apartment, begged him not to enter and risk infecting everyone. The problem for Tony was that he had nowhere to go. Lolita, sharing his story in a town hall meeting, recounts, “He did not have money to rent a hotel room. What he did was he slept in his car. When he could no longer stand it, he pitched a tent outside, at the backyard of his apartment building.” Fortunately, Tony did not have to stay outside for the entire 14 days as PWC managed to secure sufficient funds to rent an apartment for him and others displaced by COVID-19. Summarizing their dilemma, Lolita asks rhetorically, “They always tell you, ‘self-isolate, quarantine yourself.’ But the problem is—where do you do that?” Tony’s housing struggles demonstrate the manifestations of new problems arising for domestic workers during the COVID-19: joblessness and homelessness. Drawing from the virtual public meetings, we find that these problems are ones that are shared by his peers in both Los Angeles and New York.

#### Joblessness

“My employer suddenly made me leave because of COVID-19 . . . I worked for them for 10 years,” Ana shares during one of the town hall meetings a few weeks after the pandemic started ravaging New York. The experiences of Ana is not surprising. Damayan, in its informal survey with 80 caregivers in the New York Tri-State area, found that 65% of participants lost their job or faced reduced work hours. Employers are quick to terminate domestic workers because first they fear them to be a source of the virus and an endangerment to the health of older family members; second, the shift to remote offices across industries in the United States may mean they would not need their services either temporarily or permanently; and third, employers themselves may face unemployment and confront financial precarity. Employers can also terminate domestic workers without reproach as labor protection laws, including the right to severance pay, rarely extend to them due to the informal nature of their occupation (Hondagneu-Sotelo, 2001). Despite the precarity of domestic workers, the problem of termination is arguably new. This is because a “deep alliance” is said to define relations in the intimate space of the household; it is not unusual for domestic workers to stay employed in one household for multiple years and, for those caring for the elderly, stay with them until their passing (Ibarra, 2010).

During COVID-19, employers are putting greater priority for *their* personal concerns at the expense of those of domestic workers. One concrete result is the greater likelihood of termination. Consequently, the fear of joblessness is one that continuously haunts domestic workers. Jim, a domestic worker in New York, shares that having a “simple cough” or seeking medical help, which would have been unproblematic in a pre-COVID-19 situation, could now cost anyone their livelihood. Jim’s fears are not unfounded. This is because they are precarious workers whose employers have minimal liability when terminating employees. It is for this reason that employers are unlikely to be hesitant about firing domestic workers. In prioritizing their needs, employers will likely ignore the vulnerabilities elicited by termination for their workers.

While the risk of unemployment is not exclusive to domestic workers, it raises greater challenges for this group of essential workers due to their minimal access to state unemployment assistance as many are undocumented workers. A survey of Filipinx domestic workers in Los Angeles done in collaboration with PWC found that 40% are undocumented workers (Nazareno et al., 2014). In New York, Damayan found an even higher rate of undocumented workers among its constituents: Nine out of 10 are without papers. For these domestic workers, lack of access to state’s resources is “a truth they unfortunately have to navigate.” As Aquilina Soriano Versoza, executive director of PWC remarks, migrant domestic workers are “falling through the cracks in terms of receiving assistance that other people are able to access.” Lolita echoes her sentiments: “Undocumented workers contribute billions to the U.S. economy . . . They pay and file taxes. They work . . . But when it comes to state benefits, why are they not included? That is the enduring question.” Fortunately, displaced domestic workers can offset their financial precarity by turning to community-based organizations such as the National Domestic Workers Alliance, which has distributed a Care Fund to its member organizations across the country including PWC and Damayan and the Nikkei Credit Union, a progressive full-service financial institution in Southern California, which grants small loans at low interest rates to undocumented workers.

#### Homelessness

The problem of joblessness aggravates the financial immobility of domestic workers, making rent an untenable expense. To protect the public, the states of California and New York have accordingly signed executive orders to ban evictions and foreclosures. In California, tenants were additionally given a 12-month extension for paying unsettled rents. More recently, the city of Los Angeles launched an “Emergency Renters Assistance Program,” which allocated $103 million in rent subsidies to families affected by the pandemic, regardless of immigration status (City of Los Angeles Housing + Community Investment Department, 2020). Given the limited funding, however, the program could only cater to no more than 50,000 households.

As indicated by Tony’s story, it is not only joblessness that puts one’s housing security at risk, but it is also the need to self-quarantine. Isolation is made impossible by the fact that migrant workers are likely to reside in shared housing. Riya Ortiz, an organizer at Damayan explains:

That’s the struggle in our community, especially with our members—we live in basements and really cramped housing. That’s how we’re able to survive. That’s why we also don’t have homeless members because we would just cramp ourselves in tiny spaces.

Her counterpart in Los Angeles agrees. Sharing the same concerns, Lolita comments:

The problem is, many caregivers here in LA, because of expensive housing, could not afford to rent their own apartment. It’s the same in New York. So, one apartment will have six caregivers . . . How will you be able to self-isolate?

In response to this question, individuals like Tony found out that they cannot, indicating that COVID-19 has upended the coping mechanism of shared housing that migrants have developed to address the risk of homelessness they face as low-wage workers in expensive urban centers of the United States.

Progressive state officials recognize this to be a problem. In California, the state government launched a program to provide free or low-cost hotel rooms for frontline health workers in need of medical sheltering. Governor Gavin Newsom announced,

California is fighting to protect those who are protecting us . . . Health workers are the heroes of this moment. As we ramp up the workforce to meet the demand we are also stepping up to help keep our workers’ families safe by providing hotels as temporary housing options. (Office of Governor Gavin Newsom, 2020)

While PWC lauds this initiative, they complain about its inaccessibility. Lolita laments, “It is very hard to get through the phone lines—they’re always busy. Perhaps a lot of people are trying to contact them and are in need of housing.” Access to this benefit is also made difficult by the bureaucratic system put in place. If suspecting they had been exposed to the virus, health care workers, including domestic workers caring for the elderly, must prove their “positive” result to avail of the benefit, which makes it an unwieldy requirement considering that it initially took about seven to 10 days to get test results back.

In sum, echoing previous findings on the structural embeddedness of mechanisms of devaluation, we find that the new problems of joblessness and homelessness among Filipinx migrant caregivers in the time of COVID-19 are situated within broader social hierarchies and barriers that materially denigrate migrants and low-skilled workers (Glenn, 1992). First, as undocumented migrants, they lack access to state support available to other workers. They are thus left to depend on mutual aid from community advocacy groups. Asserts one community organizer: “if we cannot rely on the government, on the laws, our resource really is ourselves, our family, our neighbors.” Second, as low-skilled workers, migrant Filipinx domestic workers are made invisible. While health workers are collectively touted as “heroes,” domestic workers are rarely at the forefront of our imagination. The symbolic valorization of “essential workers” under COVID-19 does not disestablish the class- and skill-based distinctions among health care workers. As Lolita notes: “It seems that they are not considering caregivers as part of the healthcare team. It’s always the doctors, nurses, those working in the hospitals. That’s why it takes a long time to look for resources.”

The same problem of invisibility troubles domestic workers in New York, compelling the Damayan organizer Riya to share:

Many of our members are also frontliners, right? Even if we’re not officially considered essential workers—the caregivers who are caring for the sick, the domestic workers who continue to care for the children, the housekeepers who continue to clean the house of their employers. We are essential in this society—and without the labor of our members, they won’t be able to function or do their thing. Domestic work makes all work possible.

## Conclusion

This article describes and examines conditions that have arisen for domestic workers during the tumultuous period of the COVID-19 global pandemic. It asks the question: Does the symbolic celebration of essential workers, including domestic workers, as heroes extend to improved material remuneration? Domestic workers would likely respond to this latter question with a resounding “no” and claim to be nothing but “heroes without capes,” symbolically celebrated but materially disregarded. During the COVID-19 global pandemic, domestic work is considered essential but workers are not. They are arguably better described as *expendable essential workers* whose work is in demand but not fairly remunerated. Inadequate remunerations confronted by domestic workers include heightened job insecurity, absence of overtime pay, the lack of sufficient personal protective equipment, and inadequate rest due to greater labor demands.

In stark contrast to their symbolic celebration as “heroes,” domestic workers could find their welfare deprioritized during the COVID-19 pandemic. Given priority instead are the risks engendered by the pandemic on employers, whose mitigation of their economic and health insecurities usually come at the cost of the well-being of domestic workers. A tool they can utilize as a controlling mechanism in doing so is the cultural construction of domestic work as essential. The view of domestic work as essential allows employers to morally justify the sudden termination of domestic workers as it at the same time reduces the guilt potentially associated with demanding that domestic workers transition from live-out to live-in employment. The construction of domestic workers as essential also magnifies the unrequited loyalty expected by employers as it questions the deep alliances that are said to define relations in domestic work (Ibarra, 2010). Employers can feel justified to increase workplace expectations.

Overall, domestic work remains a devalued occupation, one considered “dirty work” and labeled “unskilled.” How has its categorization as “essential” transformed its valuation? Our findings demonstrate that such categorization has not necessarily resulted in the betterment of work conditions as it has instead functioned as a controlling mechanism used to extract uncompensated labor that allows the public pedestalizing of their vulnerabilities as “sacrifices.” Akin to labeling domestic workers as “one of the family,” categorizing them as “essential” potentially justifies the decommodification and reduction of the material compensation of domestic work. The potential worsening of labor conditions during the pandemic suggests the aggravation of the unequal relations that define domestic work. Indeed, COVID-19 is not an equalizer. Remaining in place are not only workplace inequities but also labor market hierarchies including those among “essential workers” as we find that medical doctors are valued but domestic workers are not.

## Notes

1 This category of labor garnered much public attention during the COVID-19 pandemic and made markedly visible in public discourse including in the news media, policy dialogues and advocacy efforts by NGOs, as well as federal government advisories and state issued executive orders.

2 As an example, see the advisory from the Centers for Disease Control and Prevention on the elderly as a high-risk group (https://www.cdc.gov/coronavirus/2019-ncov/need-extra-precautions/older-adults.html).

3 The first and third authors had been volunteering for Pilipino Workers Center and were engaged in ongoing research on the social mobility and activism of domestic workers when the pandemic hit the United States.

4 We address the organizers and members of the advocacy groups in this manner, following the common practice of using courtesy titles in the Philippines to show respect.

5 See the government website on Occupational Safety and Health Administration (https://www.osha.gov/workers/).

6 Advocates in the United States prefer to use the words “consumer” or “patient” when referring to employers in order to foreground the services provided by the domestic worker. Doing so also mitigates the unequal relationship suggested by employer and employee.
